# DOES PSYCHOLOGICAL FLEXIBILITY MEDIATE CARE-RELATED STRESS ON QUALITY OF LIFE FOR DEMENTIA CAREGIVERS?

**DOI:** 10.1093/geroni/igae098.2742

**Published:** 2024-12-31

**Authors:** Elizabeth Fauth, Ty Aller, Jacob Gossner, Audrey Juhasz, Michael Levin

**Affiliations:** Utah State University, Logan, Utah, United States; Utah State University, Logan, Utah, United States; Utah State University, Logan, Utah, United States; Utah State University, Logan, Utah, United States; Utah State University, Logan, Utah, United States

## Abstract

Caregivers of individuals with dementia experience care-related burden and stressful reactions to behavioral and psychological symptoms of dementia (BPSD) which can negatively impact multiple domains of life, including decreased global quality of life (QoL). Transdiagnostic interventions, such as those using Acceptance and Commitment Therapy (ACT) benefit dementia caregivers by addressing underlying mechanisms that impact depression, anxiety, QoL, and more. The transdiagnostic mechanism in ACT is psychological flexibility. The current study used pre-intervention (T1), post-intervention (T2) and 6-week followup (T3) data from a study of caregivers using a 6-session online, self-guided ACT program, tailored to dementia caregiving. Stepwise regression analyses (N=79) tested longitudinal mediation, examining if T2 psychological flexibility (CompACT; Francis et al., 2016), explained associations between T1 caregiver burden (Bédard et al., 2001) and T3 QoL (de Boer et al., 2004). A similar model used T1 stress reactivity of BPSD (Teri et al., 1992) as the independent variable, instead of burden. Based on correlations, both models controlled for whether the caregiver lived with the care receiver. Results indicate that psychological flexibility partially mediated the impact of burden on QoL, and fully mediated the impact of stress reactivity on QoL. Findings suggests that psychological flexibility may be an important mechanism in mitigating the impact that care-specific stress experiences have on global QoL. Caregivers often cannot leave this role, requiring them to learn to live with care-related stress in ways that do not damage their long-term wellbeing. Psychological flexibility is amenable to intervention and ACT offers promising solutions in dementia caregivers.

